# Characterization of the urinary microbiota of elderly women and the effects of type 2 diabetes and urinary tract infections on the microbiota

**DOI:** 10.18632/oncotarget.21126

**Published:** 2017-09-21

**Authors:** Fengping Liu, Zongxin Ling, Yonghong Xiao, Qing Yang, Li Zheng, Ping Jiang, Lanjuan Li, Wei Wang

**Affiliations:** ^1^ Department of Urology, The First Affiliated Hospital, College of Medicine, Zhejiang University, Hangzhou, Zhejiang, 310003, China; ^2^ State Key Laboratory for Diagnosis and Treatment of Infectious Diseases, The First Affiliated Hospital, College of Medicine, Zhejiang University, Hangzhou, Zhejiang, 310003, China; ^3^ Collaborative Innovation Center for Diagnosis and Treatment of Infectious Diseases, The First Affiliated Hospital, College of Medicine, Zhejiang University, Hangzhou, Zhejiang, 310003, China; ^4^ Nursing Department, Jiangsu Vocational College of Medicine, Yancheng, Jiangsu, 224005, China

**Keywords:** elderly women, lactobacillus, type 2 diabetes mellitus, urinary microbiota, urinary tract infection

## Abstract

Evidence shows that urine has complex bacterial profiles with considerable variation between individuals. Aging and age-related conditions can lead to the changes to the composition of urine, which means that the available nutrition for bacteria in the bladder changes with age. We explored the characteristics of the urinary microbiota of elderly women and whether these are associated with age-related conditions such as diabetes and urinary tract infections. An elderly and a non-elderly cohort of women were included. Magnetic beads were used to isolate bacterial genomic DNA, which was analyzed based on the V3-V4 hypervariable region of the 16S rRNA gene. There were significant differences between the elderly and non-elderly regarding thirteen genera of bacteria. For example, the relative abundance of *Lactobacillus* was dramatically reduced in the elderly compared with the non-elderly; it also decreased with age in the elderly cohort and it was not correlated with urine pH. The relative abundance of *Peptococcus* increased with age in the elderly while the abundance of *Bifidobacteria* decreased with age. The abundance of *Escherichia coli* was the same in the two cohorts, and it increased with water intake and was not associated with urinary tract infection events. Higher levels of *Lactobacillus* (including *Lactobacillus iners*) in the elderly were associated with diabetes, and lower levels of *Peptoniphilus* and *Dialister* were correlated with asymptomatic bacteriuria. The urinary microbiota of women is affected by ageing, type 2 diabetes mellitus and asymtomatic bacteriuria.

## INTRODUCTION

The number of people in the world aged 60 or over is projected to grow by 56% [1]. The sex ratio in 2015 among those aged 60 or over was 86 men per 100 women [1]. Elder individuals are more susceptible to infections because their immune system are weaker. Urinary tract infections (UTIs) is one of the most commonly reported infections in elder adults [2]. Several urinary defense mechanisms exist to combat uropathogens, such as low pH, extremes of osmolality, and high concentrations of organic acids, can become impaired with age [3]. Thus elderly women are more UTIsthan younger women [3]. In addition, menopause may be a risk factor for UTIs [4]. The level of free glycogen in post-menopausal women is lower than that in pre-menopausal women [5], and levels of free glycogen are associated with a vaginal microbiota that is dominated by *Lactobacillus* [6] and a low vaginal pH [7], which are both protective against uropathogens [6, 8].

The number of elderly individuals with diabetes is growing substantially [9], and over 90% of patients with diabetes have type 2 diabetes mellitus (T2DM) [10]. T2DM is not due to insufficient use of insulin but due to insufficient insulin secretion and insufficient insulin action. T2DM is more prevalent in the elderly than in younger individuals [11], for example, the prevalence in young adults in China is less than 10%, while it is 22.5% in elderly individuals [11]. Diabetes can lead to changes in the composition of the patient’s urine, including an increased level of urine glucose (UGLU) [12] and an increased pH [13], which provides a favorable microenvironment for the reproduction of harmful bacteria [14].

Generally, clinicians administer antibiotics for UTIs according to the results of standard urine cultures (SUCs) [15]. However, the results of 16S rRNA sequencing and the results of expanded quantitative urine cultures have recently shown that SUC techniques may not detect the presence of anaerobic, fastidious, intracellular and slow-growing bacteria [16–21] or bacteria that are embedded within biofilms [22–24]. Furthermore, recent evidence has shown that healthy people have resident microbiota [25–29]. Therefore, when antibiotics are administered for UTIs on the basis of SUC results, further microbial dysbiosis in the bladder can occur.

Previous studies have demonstrated the differences in the urinary microbiota of individuals with urological diseases [25, 27–39] such as urgency urinary incontinence (UUI) [28, 29, 34, 37, 38], asymptomatic bacteriuria (AB) in those with neuropathic bladder (NB) [28], and uncomplicated stress urinary incontinence [32]. Karsten et al. [29] found that, in women with UUI, bacterial diversity was negatively correlated with symptom severity, and the relative abundance of *Sphingomonadales*, *Chitinophaga*, and *Brevundimonas* significantly increased in women with UUI compared with women without UUI, while the relative abundance of *Prevotella*, *Comamonadaceae*, and *Nocardioides* decreased. Pearce et al. [37] revealed that UUI patients had higher levels of *Gardnerella* and lower levels of *Lactobacillus*. Thomas-White et al. [32] reported that bacterial diversity in women with stress urinary incontinence was correlated with body mass index (BMI) and hormonal status. Lewis et al. [25] found that the bacterial genera in urine specimens from women were more diverse than those in urine specimens from men. Moreover, the urinary microbiota of men and women who were 70 or over were dominated by *Jonquetella*, *Parvimonas*, *Proteiniphilum*, and *Saccharofermentans* [25]. However, very few elderly women participated in the study, and a larger sample size is needed to make firm conclusions about the urinary microbiota of elderly women [25].

The influence of ageing on the microbiota of humans is being increasingly recognized in the medical field [40–43]. It is reported that, compared with younger adults, elderly adults tend to have a reduced diversity of gut microbiota, a large inter-individual variability of gut microbiota, lower levels of Firmicutes, *Bifidobacteria*, *Clostridium* cluster *XIV*, and *Faecalibacterium prausnitzii*, and higher levels of Enterobacteriaceae and Bacteroidetes [44, 45].

Bacteria living in a host benefit by obtaining nutrition from the host [22]. As mentioned previously, aging and menopause can lead to the changes to the composition of urine, which means that the available nutrition for bacteria in the bladder changes with age. Moreover, age-related diseases such as T2DM also alter the composition of urine. It is hypothesized that the urine of elderly women provides a different microenvironment for the growth of microbes than that of young adults, especially if the elderly women have T2DM. Therefore, the aim of this study is to explore the characteristics of the urinary microbiota of elderly women and to examine whether they are associated with the age-related conditions T2DM and AB.

## RESULTS

### Description of cohorts

Table 1 displays the baseline characteristics of the participants in the elderly and non-elderly cohorts. There were similar levels in the two cohorts of individuals with diabetes, individuals with hysterectomies, asymptomatic bacteriuria samples, UGLU positive samples, and married, single and widowed individuals. Both cohorts also had similar mean values for BMI, water intake, urine pH and UTI events. As expected, those in the elderly cohort were more likely to be postmenstrual.

**Table 1:** Descriptive data of participants in the study

Parameter	Value for cohort (n^a^)^b^ or statistic		
	E (*n* = 50)	NE (*n* = 50)	*P*-value ^c^
Age	71.86 ± 6.70	50.06 ± 7.51	0.000
T2DM [no. (%)]	24 (48)	26 (52)	0.842
Taking metformin [no. (%)]	24 (48)	26 (52)	0.842
Marital status [no. (%)]			
Married	41 (82)	47 (94)	0.121
Single	1 (2)	1 (2)	1.000
Widowed	8 (16)	2 (4)	0.092
Menstrual status [no. (%)]			
Premenstrual	0 (0)	19 (38)	0.000
Postmenstrual	48 (96)	28 (56)	0.000
Hysterectomy	2 (4)	3 (6)	1.000
Body mass index (kg/m^2^)	23.64 ± 5.15	23.49 ± 3.36	0.867
Water intake d (ml)	2453.65 ± 833.93	2445.28 ± 747.86	0.958
Events of UTIs	0.60 ± 1.36	0.34 ± 0.69	0.229
Asymptomatic bacteriuria e [no. (%)]	7 (14)	2 (4)	0.160
Urine pH	5.83 ± 0.68	5.94 ± 0.58	0.386
Urine glucose POS [no. (%)]	5 (10)	6 (12)	1.000

^a^n, no. of subjects; ^b^Mean ± SD or no.(%); ^c^Pearson’s chi-square and Fisher’s exact tests were used with categorical variables. Independent *t*-test was used with continuous variables, *P* < 0.05 was considered statistically significant; ^d^ The amount of water intake included drinking water intake and dietary fluid intake, which was examined by the Chinese Food Frequency Questionnaire; ^e^ Asymptomatic bacteriuria was defined according to whether the cultures tested positive for *E. coli* (no tested positive for other bacteria in the present study), which was with greater than 10^5^ colony forming units /mL of *E. coli*.

### Sequencing data

To identify the microbiome in urine sample, we performed PCR to amplify the V3-V4 hypervariable regions of the 16S rRNA gene and amplicon sequencing on the MiSeq platform. The Miseq studies provided 76,695,935 raw sequences with a median read length of 2×300 and 442 base pairs. After filtering and removal of chimeric sequences, a total of 6,087,772 reads were produced from the 100 specimens, accounting for 79.38% of the valid reads with an average of 5,997,897 reads (range from 10, 975 to 124, 403) per barcoded sample for downstream analysis. The average length was 442.41 bp (range from 423 to 491).

**Figure 1:**
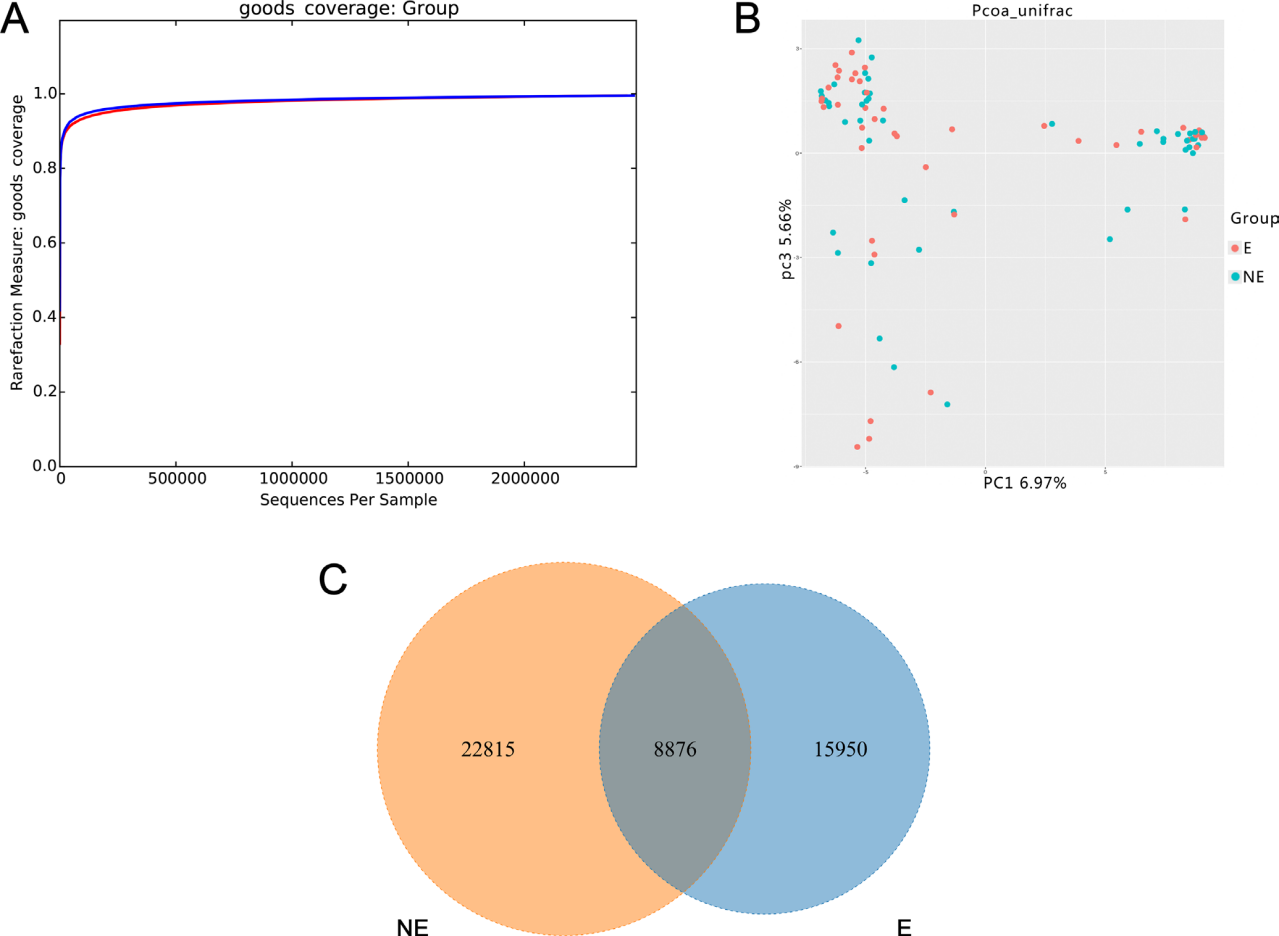
Structural comparison of urinary microbiota between two cohorts The Good’s coverage (**A**) was used to assess the sequencing depth. Principal coordinate analysis plot of the urinary microbiota based on the unweighted UniFrac metric (**B**). Venn diagram demonstrating overlap of OTUs in urinary microbiota between two cohorts (**C**). Red and blue lines and dot represent Es and NEs respectively. E and NE mean elderly female and non-elderly female respectively.

Values of Good’s coverage indicated sufficient depth for the investigation of elderly associated urinary microbiota (Figure 1A). Due to inter-individual variation, the urinary microbiota from the elderly and non-elderly individuals could not be divided into two clusters according to their composition (Figure 1B and Supplementary Figure 1). Of the 47,641 OTUs from the two cohorts, 8,876 were shared between the two cohorts (Figure 1C).

The levels of OTUs and Chao1 were slightly decreased in the elderly compared to non-elderly, while the values of the Shannon and Simpson index were slightly higher in the elderly, though no significant differences were detected (Table 2).

**Table 2:** Comparison of richness and diversity estimators between the E and NE cohort, EDM and ENDM group, EAB and ENAB group

Parameter^a^	E (*n* = 50)	NE (*n* = 50)	*P*-value ^b^	EDM (*n* = 24)	ENDM (*n* = 26)	*P*-value	EAB (*n* = 7)	ENAB (*n* = 43)	*P*-value
OTUs ^c^	2073.86 ± 1084.31	2351.60 ± 1691.51	0.331	1590.54 ± 660.78	2520.00 ± 1213.04	0.002	1373.86 ± 594.87	2187.81 ± 1107.32	0.012
Chao1	2083.26 ± 1046.69	2927.40 ± 3549.12	0.110	1805.04 ± 967.20	3754.96 ± 4629.43	0.045	1314.05 ± 379.23	2208.48 ± 1069.18	0.035
Shannon	4.82 ± 2.58	4.66 ± 2.72	0.773	4.40 ± 2.08	5.31 ± 3.19	0.241	2.91 ± 2.03	5.13 ± 2.54	0.029
Simpson	0.72 ± 0.28	0.69 ± 0.25	0.618	0.72 ± 0.24	0.72 ± 0.27	0.965	0.52 ± 0.29	0.75 ± 0.26	0.078

^a^ The parameters were calculated by QIIME software; ^b^ independent *t*-test was used to compare the value of parameters. *P* < 0.05 was considered statistically significant; ^c^ the operational taxonomic units (OTUs) were defined at the 97% similarity level.

**Figure 2:**
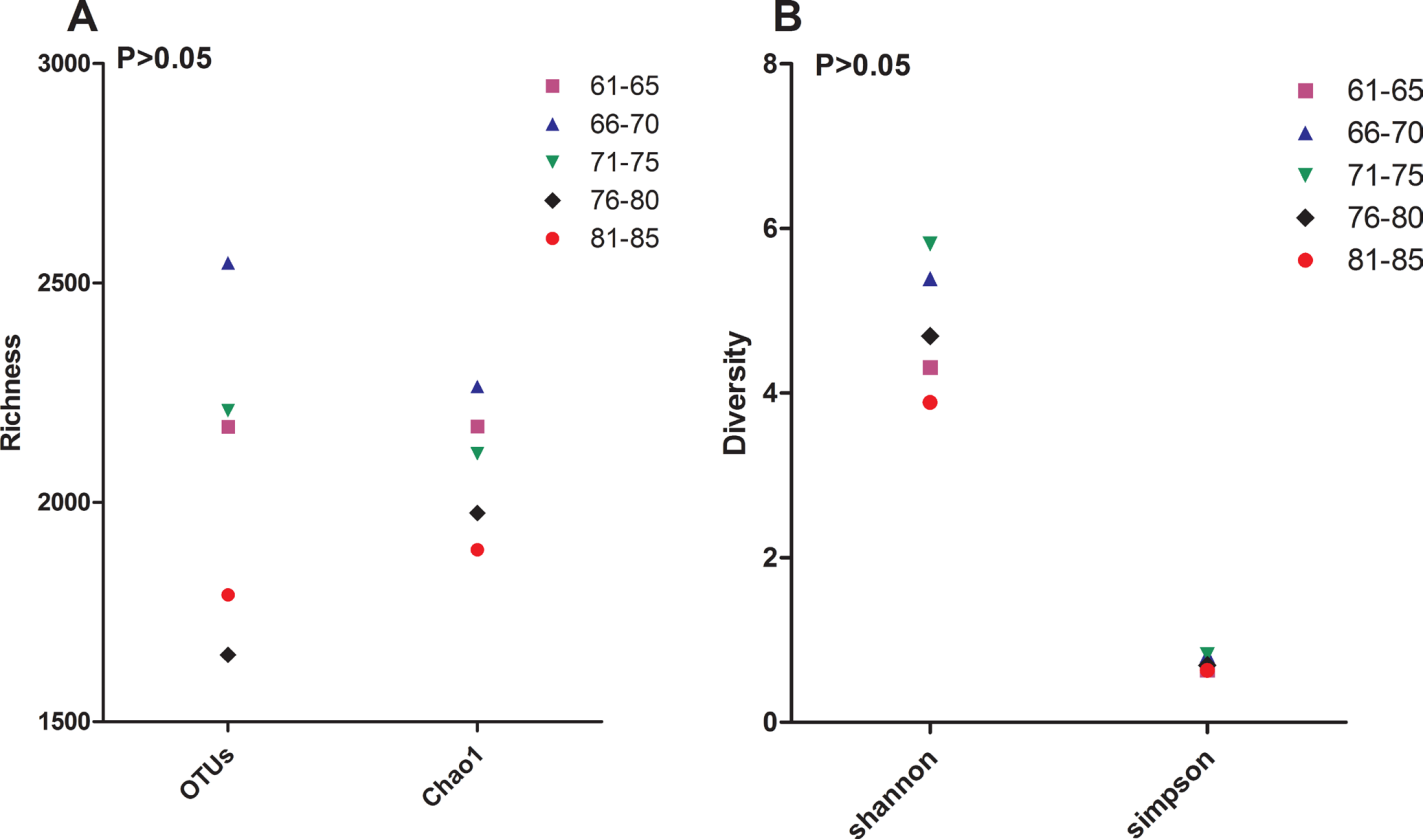
Bacterial diversity The value of OTUs, Chao1 (**A**), Shannon and Simpson (**B**) in sub-age groups in the elderly cohort. The operational taxonomic units (OTUs) were defined at the 97% similarity level; the parameters were calculated by QIIME software.

In addition, the bacterial richness and diversity decreased with age in the elderly cohort, although no significant differences were detected between the age groups (*P* > 0.05) (Figure 2A and 2B).

The richness indices were significantly higher for those in the elderly non-diabetes mellitus (ENDM) group compared with those in the elderly diabetes mellitus (EDM) group, while the diversity indices were slightly higher for those in the ENDM group compared with those in the the EDM group (Table 2). Both the indices of richness and diversity for the participants in the elderly with asymtomatic bacteriuria (EAB) group were significantly lower than those for the participants in the elderly non-asymtomatic bacteriuria (ENAB) group, except for the Simpson index (Table 2).

### Difference in urinary microbiota between the elderly and non-elderly subjects

At the bacterial phylum level, the samples from the elderly individuals were dominated by Proteobacteria (56.05%), Firmicutes (19.12%), Bacteroidetes (14.54%), Actinobacteria (5.99%), and Thermi (1.05%). The samples from the non-elderly individuals were largely characterized by Proteobacteria (57.67%), Firmicutes (20.39%), Bacteroidetes (9.49%), Actinobacteria (8.62%), and Acidobacteria (0.95%).

**Figure 3:**
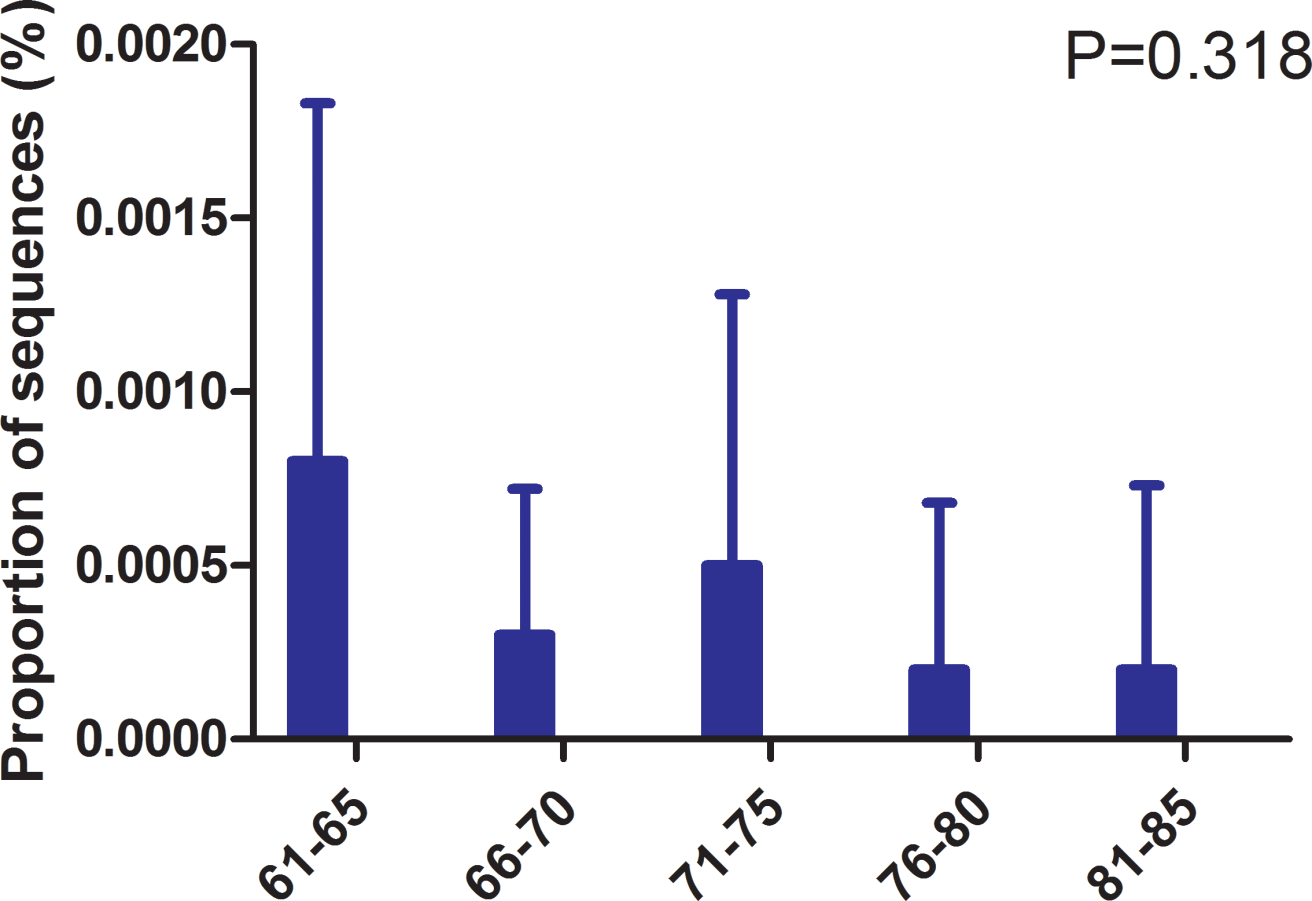
Relative abundance of *Lactobacillus* in age subgroups The relative abundance of *Lactobacillus* in age subgroups in the elderly cohort was compared. *P*-values were based on ANOVA test and corrected for multiple comparisons.

When the bacterial genus level analyzed, we found that the samples from the elderly and non-elderly cohorts contained 373 and 385 genera, respectively. *Prevotella* (16.66%), *Bacteroides* (8.69%), *Lactobacillus* (6.04%), *Pseudomonas* (4.83%), and *Acinetobacter* (3.65%) were the predominant bacteria in the elderly cohort, while the samples from the non-elderly cohort were dominated by *Prevotella* (14.23%), *Lactobacillus* (13.22%), *Bacteroides* (5.94%), *Pseudomonas* (4.58%), and *Acinetobacter* (4.00%). Totally, there were significant differences in the relative abundance of 13 genera between the elderly and non-elderly cohorts (Table 3). The relative abundance of *Lactobacillus* was significantly lower in the elderly cohort, and it was lower in the 81–85 age group compared with the 61–65 group, although it was higher in the 71–75 group compared with the 66–70 group (Figure 3). *Lactobacillus* could not be detected in the samples from three participants in the elderly cohort who were aged over 76. The relative abundance of *Lactobacillus* was not significantly associated with the pH of the elderly women urine (r = 0.073, *P* = 0.470). Interestingly, *Peptococcus* was not found in the 61–65 year group, although it was present in 20% (2/10) of the samples from the 66–70 group and 33.33% (10/30) of the samples from the 70 plus group. The relative abundance of *Bifidobacteria* was lower in the elderly cohort compared with the non-elderly cohort, although the difference was not significant. Interestingly, there was a negative correlation between the relative abundance of *Bifidobacteria* and age (r = −0.34, *P* = 0.016).

**Table 3:** The relative abundance of bacteria at genus level in groups

Taxon	E (*n* = 50)	NE (*n* = 50)	*P*-value	Taxon	EDM (*n* = 24)	ENDM (*n* = 26)	*P* -value	Taxon	EAB (*n* = 7)	ENAB (*n* = 43)	*P* -value
Lactobacillus	6.04 ± 18.33	13.22 ± 20.52	0.001	Aeromonas	0.44 ± 2.04	2.47 ± 10.57	0.011	Peptoniphilus	0.01 ± 0.02	0.02 ± 0.03	0.012
Sneathia	0.45 ± 1.39	1.88 ± 4.00	0.001	Agrobacterium	0.03 ± 0.08	0.18 ± 0.63	0.008	Dialister	0.01 ± 0.01	0.02 ± 0.02	0.016
Shuttleworthia	0.36 ± 1.00	2.12 ± 5.48	0.040	Anaeromyxobacter	0.06 ± 0.24	0.05 ± 0.09	0.017				
Bacillus	0.51 ± 0.83	2.24 ± 7.44	0.033	Bdellovibrio	0.01 ± 0.03	0.01 ± 0.02	0.012				
Gemella	0.01 ± 0.05	0.20 ± 0.58	0.004	Bilophila	0.04 ± 0.10	0.12 ± 0.18	0.048				
Bdellovibrio	0.01 ± 0.03	0.11 ± 0.34	0.005	Butyricimonas	0.01 ± 0.03	0.07 ± 0.09	0.004				
Sphingobium	0.03 ± 0.07	0.02 ± 0.07	0.012	Clostridium	0.18 ± 0.26	0.46 ± 0.51	0.029				
Hydrogenophaga	0.01 ± 0.04	0.05 ± 0.13	0.039	Desulfovibrio	0.04 ± 0.09	0.09 ± 0.12	0.026				
Proteus	0.01 ± 0.03	0.04 ± 0.12	0.036	Eggerthella	0.05 ± 0.22	0.04 ± 0.10	0.033				
Geobacillus	0.02 ± 0.07	0.12 ± 0.62	0.031	Enterobacter	0.02 ± 0.05	0.32 ± 0.67	0.039				
Novosphingobium	0.01 ± 0.03	0.03 ± 0.06	0.019	Enterococcus	0.21 ± 0.60	1.06 ± 3.76	0.026				
Bosea	0.01 ± 0.03	0.00 ± 0.01	0.030	Erwinia	0.03 ± 0.09	0.63 ± 1.24	0.014				
Catenibacterium	0.01 ± 0.02	0.01 ± 0.03	0.031	Fusobacterium	0.59 ± 2.52	0.85 ± 0.94	0.001				
				Hydrogenophaga	0.01 ± 0.05	0.01 ± 0.02	0.028				
				Klebsiella	0.07 ± 0.19	4.83 ± 11.91	0.001				
				Lachnobacterium	0.01 ± 0.04	0.07 ± 0.11	0.002				
				Lysobacter	0.05 ± 0.15	0.40 ± 1.62	0.006				
				Microbacterium	0.07 ± 0.20	0.01 ± 0.02	0.017				
				Mitsuokella	0.01 ± 0.04	0.02 ± 0.03	0.012				
				Modestobacter	0.18 ± 0.90	0.01 ± 0.03	0.020				
				Nitrospirae	0.14 ± 0.37	1.03 ± 1.50	0.014				
				Odoribacter	0.03 ± 0.10	0.12 ± 0.18	0.024				
				Parvimonas	0.38 ± 0.89	0.31 ± 0.73	0.036				
				Phascolarctobacterium	2.19 ± 4.25	2.90 ± 2.59	0.029				
				Ramlibacter	0.01 ± 0.02	0.03 ± 0.05	0.009				
				Rhodoplanes	0.01 ± 0.02	0.04 ± 0.08	0.038				
				Stenotrophomonas	0.07 ± 0.18	0.37 ± 1.25	0.041				
				Thiobacillus	0.01 ± 0.03	0.01 ± 0.03	0.022				
				Turicibacter	0.01 ± 0.02	0.00 ± 0.00	0.022				

The Mann-Whitney *U*-test was used to compare the relative abundant difference in two groups. *P* < 0.05 was considered statistically significant.

Interestingly, when the bacterial species level was examined, we noted that the relative abundance of *Escherichia coli* did not differ between the elderly and non-elderly cohorts (0.004 ± 0.004 vs. 0.004 ± 0.004, *P* = 0.775).

### Factors associated with urinary microbiota in the elderly cohort

#### T2DM affected elderly urinary microbiota

Those in the EDM group had significantly lower levels of Nitrospirae, Verrucomicrobia, and Planctomycetes compared with those in the ENDM group at bacterial phylum level (data not shown). Samples from the EDM group contained 283 genera while samples from the ENDM group contained 313 genera. The dominant bacteria in the EDM group were *Prevotella* (20.74%), *Lactobacillus* (10.78%), *Bacteroides* (8.29%), *Acinetobacter* (5.78%), and *Halomonas* (1.87%), while the ENDM group was dominated by *Prevotella* (12.89%), *Bacteroides* (9.07%), *Pseudomonas* (8.48%), *Faecalibacterium* (3.31%), and *Porphyromonas* (2.95%), and *Lactobacillus* only accounted for 1.66% of the sequences. In addition, there were significant differences in the relative abundance of 28 genera between the EDM and ENDM groups (Table 3). *Lactobacillus* had a declined trend in T2DM patients with FBG > 10 mmol/l than FBG ≤ 10 mmol/l, and decreased in the urine glucose positive samples comparing to the negative samples (Supplementary Figure 2A and 2B). The relative abundance of *Lactobacillus iners* was much greater in the EDM group compared with the ENDM group (0.05 ± 0.01 vs. 0.00 ± 0.00, *P* = 0.036). However, it was not associated with FBG levels (r = −0.198, *P* = 0.354) and there was no significant difference between the UGLU positive and UGLU negative samples (0.00 ± 0.00 vs. 0.06 ± 0.02, *P* = 0.273).

### Elderly urinary microbiota associated with AB

Those in the EAB group had significantly lower levels of Firmicutes, and significantly higher levels of Fusobacteria than those in the ENAB group (data not shown). The samples from the EAB group contained 151 genera, while samples from the ENAB group contained 348 genera. Furthermore, the levels of *Peptoniphilus* and *Dialister*, were significantly lowered in the EAB group compared with those in the ENAB group (Table 3). Interestingly, *Escherichia coli* was not associated with UTI events (r = 0.43, *P* = 0.765), but it increased with water intake (r = 0.412, *P* = 0.003).

### BMI correlated to elderly urinary microbiota

In the elderly cohort, the relative abundance of Firmicutes increased with BMI, from those with normal weight (18.5 ≤ BMI < 23) to those who were overweight (23 ≤ BMI < 25), to those who were obese (BMI ≥ 25) [58] (0.15 ± 0.15 vs. 0.18 ± 0.19 vs. 0.42 ± 0.16, *P* = 0.002).

## DISCUSSION

The urinary microbiota of elderly women showed similar inter-individual variation to that of the non-elderly individuals, suggesting that other factors affect microbiota besides age. The Venn diagram shows that only 18.63 % of the OTUs were shared between the elderly and non-elderly individuals, suggesting that urinary microbiota change with age. The richness and diversity of the microbiota were slightly lower in the elderly cohort compared to the non-elderly cohort, and it generally decreased with age in the elderly cohort. A similar trend was reported in a previous study, that is, women who were over 70 had 75% fewer bacteria than women who were aged 20–49 [25]. Notably, the number of UTI events and asymptomatic bacteriuria samples in the elderly cohort were higher than in the non-elderly cohort in our study. This suggests that the lower bacteria richness and diversity in the elderly women is related to UTIs.

It seems the distribution of urinary microbiota in Chinese female is different from population from other countries. It might be due to dietary habits in China are not the same as western countries. Previous studies demonstrated that intestinal microbiota composition differed markedly according to dietary intake [46], and diet could induce differences in microbitoa composition and microbial metabolites, including volatile fatty acids, secondary bile acids, and products of protein degradation [47]. Thus urinary microbiota might be indirectly influenced by intestinal microbiota alterations caused by dietary pattern, that is, the composition of urine is influenced by nutrient metabolism [48].

A previous study has demonstrated that the abundance of Proteobacteria was significantly higher in patients with UTIs than in controls [39], while the levels of Proteobacteria tended to be lower in the elderly compared with the non-elderly in our study. Although it is well recognized that elderly women have a higher prevalence of UTIs than younger women, there was no significant difference in AB between the elderly and non-elderly subjects recruited in the present study. A study involving a large sample of elderly women with UTIs is needed to determine whether a lower relative abundance of Proteobacteria plays a role in reducing UTIs in elderly women. In addition, the relative abundance of Firmicutes was significantly associated with BMI in the elderly cohort, which is similar to the findings of a study on the gut microbiota of obese mice [49]. This suggests that an individual’s metabolism, including their energy conversion efficiency, may be affected by the relative abundance of Firmicutes in their urine.

As expected, the relative abundance of *Lactobacillus* was dramatically lower in the elderly cohort compared with the non-elderly cohort, and it was not detected in several of the samples of elderly individuals. Similar findings were reported by a recent study that showed that *Lactobacillus* predominated in premenopausal women but not in postmenopausal women [29]. Although the relative abundance of *Lactobacillus* in the elderly tended to decline with age, the trend fluctuated, ie, the abundance in those aged 71 to 75 was slightly higher than that in those aged 66 to 70. This suggests that estrogen plays a limited role in regulating the reproduction of *Lactobacillus* or that the decrease estrogen with age may plateau in elderly women. The plasma content of estrogen drops from about 129 ng/L in the reproductive years to about 18 ng/L after menopause, affecting the vagina [50]. The vaginal epithelium contains estrogen-alpha receptors which might deplete the cell densities of Lactobacilli after menopause [51]. Similar result might be present in the urinary bladder, since a previous study reported that estrogen receptors alpha could be detected in the mucosa of the bladder [52]. It is worth noting that a linear relationship was not found between the pH of the urine and the relative abundance of *Lactobacillus*, which suggests that clinicians should reconsider the effectiveness of using *Lactobacillus* to lower urine pH to reduce the risk of UTIs in elderly women.

*Sneathia* and *Gemella*, which are found in patients with UUI [37], sharply decreased in the elderly individuals. The relative abundance of *Proteus*, which has been found to be enriched in patients with NB [28], was lower in the elderly individuals compared with the non-elderly individuals. The low abundance of *Sneathia*, *Gemella*, and *Proteus* may be due to the exclusion of women with UUI and NB from the study. *Peptococcus* was only detected in subjects aged over 66. Lewis et al. reported that *Peptococcus* could only be found in men over 70 years [25]. Thus the high levels of *Peptococcus* in the elderly may be associated with aging in both women and men. The abundance of *Bifidobacterium*, which was shown to be associated with young people in a study on intestinal microbiota [53], also decreased with age.

*E. coli* is the most common urinary tract pathogen, and it is responsible for 76.6% of UTIs in elderly women [54]. However, the abundance of *E. coli* was the same in the elderly and non-elderly cohorts, and it was not affected by UTI events.

EDM appeared to lower the bacterial richness and diversity, which was similar to the findings of previous studies [55]. It is unclear why the EDM group had a lower relative abundance of Nitrospirae compared with the ENDM group, given that a previous study only found Nitrospirae in patients with interstitial cystitis [30]. These results suggest that alterations in urinary microbiota are related to certain health conditions. It is worth noting that the urinary microbiota of elderly women was profoundly affected by diabetes. For example, the relative abundance of *Lactobacillus* was higher in the EDM group than in the ENDM group. This finding is inconsistent with a study on patients with UUI, which showed that the patients had a lower abundance of *Lactobacillus* than the controls [37]. However, the finding was linked to another study in which the abundance of *Lactobacillus* was higher in interstitial cystitis patients compared with controls [30]. Interestingly, compared with controls, patients with diabetes have also been shown to have more *Lactobacillus* in their gut [56]. It appears that *Lactobacillus* in urine is also affected by the onset of diabetes. Furthermore, the severity of diabetes might influence the productivity of *Lactobacillus* in urine, since the subjects with FBG > 10 mmol/L and urine glucose positive results had lower proportion of *Lactobacillus*. A recent study demonstrated that *Lactobacillus casei* CCFM419 could protect mice against diabetes involving gut microbiota, in which the levels of the inflammatory markers such as tumor necrosis factor-ɑ and interleukin-6 decreased while intestinal glucagon-like peptide-1 levels increased [57]. Future study need explore whether *Lactobacillus* in urine regulate the glucose level via the similar mechanism to intestine. Moreover, the relative abundance of *Eggerthella* was greater in the EDM group than in the ENDM group, and a study of Chinese patients reported that intestinal *Eggerthella* was linked with T2DM [58]. The abundance of *Fusobacterium* was lower in the EDM group, although it has previously been shown to increase in patients with UUI [37]. A lower abundance of *Klebsiella* was also associated with EDM, which is in line with the findings from a previous study that showed that *Klebsiella* is a uropathogen found in patients with diabetes [59]. Notably, *L. iners*, which is strongly associated with bacterial vaginosis [60], predominated in the EDM group. Additionally, it seems that the abundance of *L. iners* was neither affected by the FBG values nor by the UGLU values. This is inconsistent with the findings of a recent study on gut microbiota, in which the levels of FBG in mice decreased when they were treated with *Lactobacillus plantarum* SCS2, and treatment with *Lactobacillus casei* Zhang protected the mice against the onset of T2DM [61]. This suggests that different species of *Lactobacillus* have different effects on regulating diabetes or that *Lactobacillus* in urine plays a different role from intestinal *Lactobacillus*.

Also, having AB dramatically influenced the bacterial diversity in the elderly individuals. Those in the EAB group had less than 50% of the bacterial genera of those in the ENAB group (151/348), which is similar to the findings of a previous study [39]. It has been reported that bacterial diversity is negatively associated with the overgrowth of the more aggressive species of residential bacteria [62]. Therefore, the low bacterial diversity in the EAB group may be caused by the overgrowth of pathogenic bacteria in the bladder, which inhibits the reproduction of other bacteria. A decrease in the relative abundance of Firmicutes was linked to the EAB group, which contrasts with the findings of a previous study that found no difference between UTI patients and non-UTI participant [39]. Meanwhile, a larger abundance of Fusobacteria was associated with the EAB group, which is inconsistent with the findings of a previous study in which Fusobacteria were found to be one of the dominant bacterial phyla in women without UTIs [26]. In addition, Nienhouse et al. reported that *Dialister* was enriched in their urine culture negative samples [34], which is not similar to the result of our study that showed that the abundance of *Dialister* was significantly reduced in the EAB group. A recent study demonstrated that patients with NB had an increase in the abundance of *L. iners* [28]*.* This suggests that the role of *L. iners* should be considered when administering microbiota-based therapy for UTIs. Our study shows that the relative abundance of *E. coli* increased with water intake, which leads to questions concerning the traditional strategy of drinking water to prevent *E. coli* UTIs [63].

Urine samples are at risk of contamination by vaginal and gut bacteria [27] so suprapubic aspiration (SPA) has been suggested as a method of collecting samples for urinary microbiota studies [64]. However, SPA is very invasive and therefore inapplicable for use in a large study of women who have not had voiding disorders or urinary diseases. As SPA and transurethral catheter (TUC) urine specimens have similar profiles [65], we modified the TUC technique for use in our study. Additionally, in order to obtain genuine midstream urine (MSU), we used a four-tube collection method, which allowed the collection of sufficient amounts of MSU that did not come into contact with the opening of vagina and/or anus.

In the pre-experimental study, we tried techniques used in previous urinary microbiota studies, such as using a Qiagen’s DNeasy Blood & Tissue Kit with added sterile phosphate-buffered saline [34], DNA stabilization buffer [34], and phenol-chloroform-isoamyl alcohol (with or without bead beating) [28]. However, we failed to extract sufficient DNA for sequencing because of the low bacterial load in the urine. Subsequently, we isolated the DNA using a magnetic beads-based DNA extraction method with minor modifications [66], which included adding lysis buffer.

The major limitation of this study was the participants’ urine samples were not all collected at an identical room temperature during the same season, so the temperature-related differences in the rates of bacterial reproduction [67] during the process of urine collection may have influenced the urinary microbiota.

To our knowledge, this is the first study to characterize the urinary microbiota of elderly women. The diversity and composition of the microbiota of elderly women is different from those of non-elderly women, and the differences are linked to the participant’s characteristics. Additionally, age-related conditions profoundly affect the microbiota.

## MATERIALS AND METHODS

We used an individually matched case-control design, with one non-elderly woman (aged 28–60) for each elderly woman (aged 61–85) [68]. Furthermore, the individuals in the elderly cohort were selected using stratified sampling. Specifically, ten women were selected from each of the following age groups: 61–65, 66–70, 71–75, 76–80, and 81–85. The subjects were volunteers and they were recruited when they visited the First Affiliated Hospital, School of Medicine, Zhejiang University or the health service center in the local community from June 28th 2015 to January 2nd 2016. Those with diabetes had been diagnosed previously by physicians. Written Informed consent was obtained from participants, with approval of the Ethics Committee of the First Affiliated Hospital (Reference number: 295). The following criteria, as determined by participant’s medical records and/or complaints, were used to exclude subjects: UTI in the previous month; antibiotic use in the previous 3 months [69]; unable to complete the questionnaire; menstruation; urinary incontinence; known anatomic urinary tract abnormalities (e.g. cystoceles, hydronephrosis, renal atrophy or NB) [70]; urinary catheter [70].

The first urine of the day was collected. Before urine sampling, the participants were instructed to use the modified midstream urine (MMSU) collection technique which was composed of disinfection techniques and four-tube collection methods (Supplementary: Protocol 1). The MMSU procedure was supervised by a senior nurse. The tubes were immediately placed on ice and transferred to lab. Clinical laboratory criteria to define AB were the presence of ≥ 10^5^ CFU/mL of the same bacterial strain in two consecutive MMSU specimens [70]. If AB was confirmed by SUC, bacterial sequencing involving a second urine sample was carried out. The samples were coded with an anonymous research identification number. In addition, fasting blood glucose (FBG) was measured on the same day as urine sample collection. Elderly T2DM patients were divided into two subgroups: FBG ≤ 10 mmol/L group (well controlled group) and FBG > 10 mmol/L group (poor controlled group) [71], and urine glucose negative and positive group. A self-report questionnaire was used to collect demographic characteristics and water intake [72].

Samples were given anonymous identification code, which were transferred immediately to the laboratory and stored at –80°C until DNA extraction [66, 73]. The method of magnetic beads isolation of genomic DNA from bacteria was based on the manufacturer’s protocol with minor modifications (lysis buffer was added before bacterial extraction (Supplementary: Protocol 2) [66]. The 16S rRNA gene V3-V4 regions was PCR-amplified from microbial genome DNA using primers (forward primer, 5′-ACTCCTACGGGAGGCAGCAG-3′; reverse primer, 5′-GGACTACHVGGGTWTCTAAT-3′). Negative DNA extraction controls were amplified and sequenced as contamination controls. The amplicons were normalized, pooled and sequenced on the Illumina MiSeq instrument using a 300×2 V3 kit. Bacterial DNA was detected in the samples from all but one elderly participant; this participant was replaced by another one who had similar demographic characteristics.

Sequence reads processing was performed using QIIME (version 1.9.0) [74] and included additional quality trimming, demultiplexing, and taxonomic assignments. Profiling of predictive urinary microbiota was analyzed by using PICRUSt based on the Greengenes database as of 13 August 2013 [75]. Specifically, the processed sequences were subjected to subsampled open-reference operational taxonomic unit (OTU) picking against Greengenes, clustering unmatched reads with 97% identity into OTUs. QIIME was used to calculate the unweighted UniFrac distances, Shannon-Weiner indices, and phylogenetic (alpha) diversity. Principal coordinate analysis and alpha rarefaction plots were generated using QIIME. The diversity and richness of the bacteria in the urine samples were calculated using several estimates. These consisted of the level of OTUs, which provides a measure of bacterial richness) [76], Chao1 (which is also an estimate of bacteria richness) [77] and the Shannon and Simpson indices (which are measures of bacterial diversity) [78]. Hierarchical clustering was performed, and a heatmap was generated using a Spearman’s rank correlation coefficient as a distance measure and a customized script developed in the R statistical package. The output file was further analyzed using Statistical Analysis of Metagenomic Profiles software package (version 2.1.3). The sequence data from this study are deposited in the GenBank Sequence Read Archive with the accession number SRP 087709.

Statistical analysis was performed using the SPSS data analysis program (version 21.0). For continuous variables, independent *t*-test and Mann-Whitney *U*-test were applied. For categorical variables between groups, using either the Pearson chi-square or Fisher’s exact test, depending on assumption validity. For taxon among groups, ANOVA test was applied (Tukey-Kramer was used in Post-hoc test, Effect size was Eta-squared) with Storey’s false discovery rate correction approach [66]. White’s nonparameteric *t*-test was applied with Benjamini-Hochberg FDR false discovery rate correction approach [79]. All tests of significance were two sided, and *P* < 0.05 was considered statistically significant.

## SUPPLEMENTARY MATERIALS FIGURES


