# Supplementary material for: Characterization of the urinary microbiota of elderly women and the effects of type 2 diabetes and urinary tract infections on the microbiota
Source: Oncotarget. 2017 Sep 21;8(59):100678–90. doi: 10.18632/oncotarget.21126 (PMC5725054; doi:10.18632/oncotarget.21126)
Supplement: Supplementary file 1 [file oncotarget-08-100678-s001.pdf]

Characterization of the urinary microbiota of elderly women and the effects of type 2 diabetes and urinary tract infections on the microbiota

SUPPLEMENTARY MATERIALS

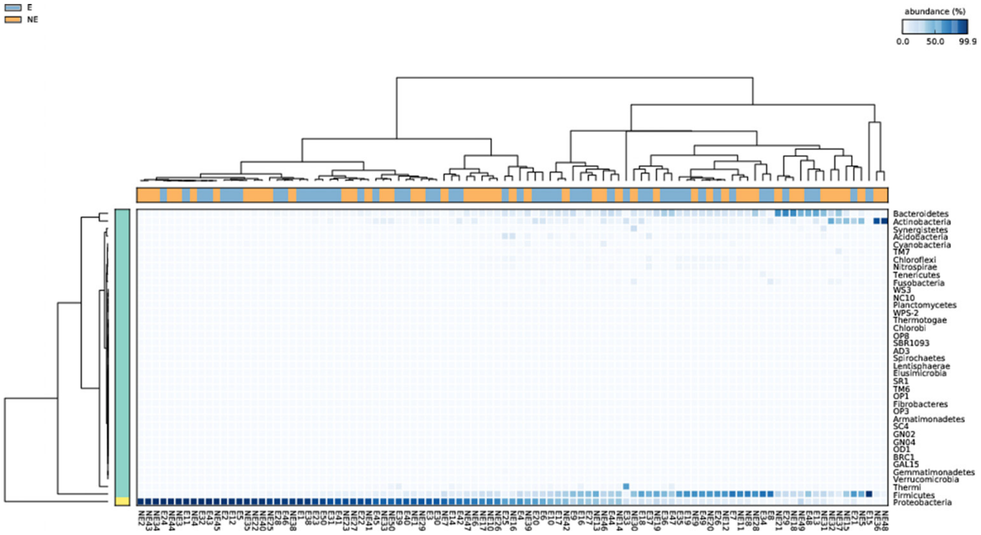

Supplementary Figure 1: Heatmap at phylum level. Hierarchical clustering was performed, and a heatmap was generated using a Spearman's rank correlation coefficient as a distance measure, as well as a customized script developed in the R statistical package. The heatmap was based on log (2) transformed values of read counts for OTUs in the two cohorts.

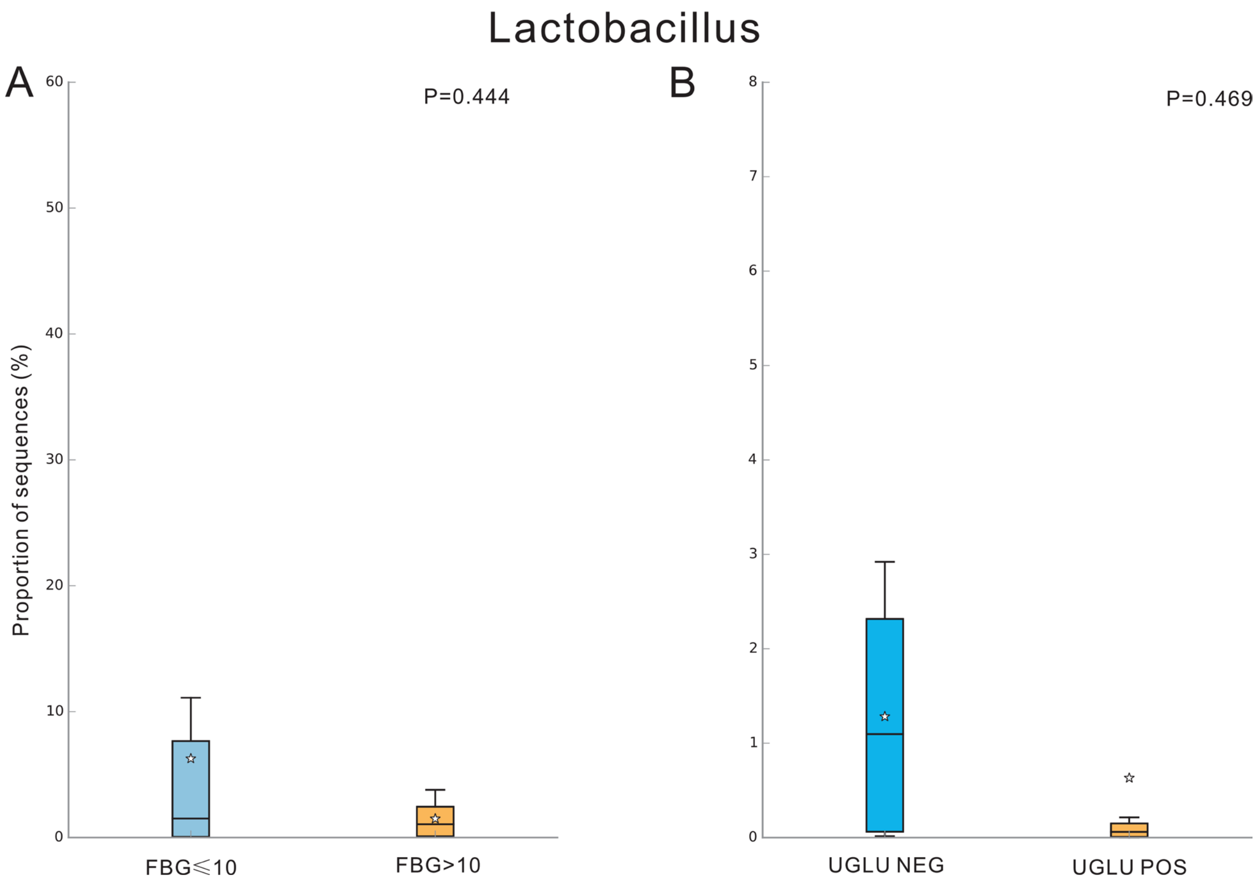

Supplementary Figure 2: The relationship between the relative abundance of Lactobacillus and FBG level and UGLU results. (**A**) Box plot showing the distribution in the proportion of Lactobacillus assigned to samples from elderly T2DM patients with FBG ≤ 10 mmoL/L, and patients with FBG > 10 mmoL/L. (**B**) Box plot showing the distribution in the proportion of Lactobacillus assigned to samples from elderly T2DM patients with UGLU NEG, and patients with UGLU POS. The median value is shown as a line within the box and the mean value as a star. Welch's *t*-test was applied, and Storey's FDR was used as a correction approach to control the false discovery rate. *P* (corrected) < 0.05 was considered significant. FBG represented fasting blood glucose.

Supplementary Protocol 1

(a) opening of Pvp-I Antiseptic (Dian'erkang, Shanghai, China) and poured onto the sterile cotton balls which were placed in a 40 mL sterile sputum cup; (b) open the four 50 mL sterile centrifuge tubes which were labeled with numbers 1, 2, 3 and 4, respectively (Tube 1, Tube 2, Tube 3 and Tube 4), and placed the interior of the cover upwards, not allowing participants to touch the interior and the edge of the tube and the cover during this procedure; (c) pulled pants down to the knees and squat on a wash bowl or a squat toilet pan, with spread legs; (d) disinfected thumb, middle and index finger of both hands with Pvp-I Antiseptic twice; (e) used the dominant hand to pick up a cotton ball, cleaned the far labial fold, starting from above the meatus down towards the rectum. Discarded cotton ball without crossing the sterile field where it was disinfected. Repeated cleaning near labial fold and down center of meatus, hold labia apart to prevent labia minora from falling back over meatus. After disinfection, participant initiated voiding the urine into Tube 1, until it reaches one-half tube. Then, without stopping the flow of urine, void urine into Tube 2, Tube 3 and Tube 4 in order. The urine stream was not permitted to stop, and the redundant was voided to the wash bowl or squat toilet. The only difference between Tube 2 and the others was that this tube should be filled at the labeled line which represents 40 mL of urine. After training, we asked the participant demonstrating the MMSU technique to assess their competency to perform the procedure.

Supplementary Protocol 2

40 mL urine was aspirated from Tube 2 and Tube 3, separated into three sections and injected into three 15 mL sterile centrifuge tubes. Each of them was pelleted by centrifugation at 4.000×g for 15 min at 4°C. Ten mL of the supernatant was decanted and the pellet was obtained by centrifugation for 15 min at 4.000×g at 4°C. The pellet was injected into a 2 mL sterile centrifugation tube which contained 500 μL of lysis buffer, which was composed of 1M Tris-HCl (pH 8.03), NaCl, 0.5M EDTA (pH 7.97), and SDS. The tube was kept at −80°C until DNA extraction. The tube was placed in liquid nitrogen for 1 min, and transferred into a water bath at 65°C for 5 min, with vigorous mixing. This last process was repeated three times with a final maintenance in the water bath for 30 min. 50 μL Agencourt AMPure XP (Beckman Coulter, USA) was added to 100 μL of the urine pellet, vortexed for 30 sec and incubated for 5 min at room temperature. The tube was placed into a magnetic separator for 5 min, and DNA was bound to magnetic beads which were drawn to the wall of the microcentrifuge tube. The supernatant was carefully removed without disrupting the magnetic beads. The sample was washed twice with 200 μL 80% ethanol for 30 sec, being placed on a magnet separator between each washing. The purified DNA was eluted with 50 μL ddH2O for 1 min. The beads, now released from the DNA, were collected with the magnet, The DNA-containing supernatant was transferred to a clean tube. The concentration of extracted DNA was determined by using a NanoDrop ND-1000 spectrophotometer (Thermo Electron Corporation, USA); its integrity and size were checked by 1.0% agarose gel electrophoresis containing 0.5 mg/mL ethidium bromide. The DNA complex was placed at −20°C until PCR amplification. All crucial steps during sample processing, DNA isolation and the entire PCR set up were performed in a laminar air flow bench, illuminated with a UV lamp prior to use in order to avoid possible contaminants.
